# A new insight on genetic diversity of sweet oranges: CAPs-SSR and SSR markers

**DOI:** 10.1186/s43141-022-00393-6

**Published:** 2022-07-14

**Authors:** Narineh Shahnazari, Zahra Noormohammadi, Masoud Sheidai, Fahimeh Koohdar

**Affiliations:** 1 Department of Biology, Science and Research Branch, Islamic Azad University, Tehran, Iran; 2 Department of Plant Sciences and Biotechnology, Faculty of Life Sciences and Biotechnology, Shahid Beheshti University, Tehran, Iran

**Keywords:** Sweet orange, SSR, CAPS-SSR, Synonym, Homonym, Homoplasy

## Abstract

**Background:**

Citrus species are among the most important and widely consumed fruit trees in the world and are subjected to increasing global cultivation. Sweet orange (*Citrus sinensis* L. Osbeck) is one of 30 species of citrus which is cultivated in different regions of Iran. In this study, 80 trees of 13 sweet orange cultivars of Mazandaran province were studied for genetic diversity and fingerprinting by five short simple repeat (SSR) marker.

**Results:**

The studied cultivars showed a high degree of genetic variability with an average genetic polymorphism of 98.46%. Behshahr and Jadeh Ghadim2 genotypes had the highest and lowest values in Nei genetic diversity, number of effective alleles, and Shannon index, respectively. Based on k-means clustering, the studied genotypes were divided into two main different groups. The high magnitude of genetic similarity between replicates of different cultivars indicated a potential case of homonymy or synonymy. DAPC analysis showed genetic admixture among some of the cultivars. The heatmap plot illustrated the alleles involved in cultivar differentiation. The CAPs analysis of monomorphic alleles of SSR loci indicated that these alleles differ in their sequences which add up to the genetic variability of citrus germplasm.

**Conclusion:**

In general, SSR markers, due to their codominant nature and abundance in genome, are a good indicator for cultivar fingerprinting and hybrid prediction in orange cultivars. The present results showed the high diversity of sweet orange trees in different cultivars in the north of the country.

**Supplementary Information:**

The online version contains supplementary material available at 10.1186/s43141-022-00393-6.

## Background

Citrus is one of the most important and abundant fruit crops in the world [20], with over the approximately 157 million tons of production in 159 countries until 2019 (http://www.fao.org/faostat/en/#data/SC).

Citrus belongs to the Citrineae subtribe of the Aurantioideae subfamily, which is one of the seven subfamilies of Rutaceae family, and it consists of two tribes (Clauseneae and Citreae), six subtribes, and 33 genera [32].

Citrus fruits are widely grown in areas with tropical, subtropical, and borderline subtropical/temperate climates [2]. The exact origin of many citrus species is not well known, but Southeast Asia is considered to be its source [22].

Citrus phylogeny and taxonomy are complicated due to the occurrence of bud mutations, sexual compatibility between citrus and related genera, wide dispersion, and long history of cultivation [18].

Climatic conditions especially in the southern and northern provinces of Iran create suitable conditions for citrus production [8]. Therefore, in 2019, Iran is ranked as the 10th largest producer of citrus in the world (http://www.fao.org/faostat/en/#data/SC). However, little is known about the genetic diversity of the Iranian citrus germplasm [6, 8, 9, 20].

Among citrus species, sweet oranges (*Citrus sinensis*) with 2*n* = 2× = 18 [20] are very popular among citrus fruits due to their many properties, such as their ability to prevent atherosclerosis, cancer, kidney stones, stomach ulcers, cholesterol levels, and high blood pressure [23].

In order to identify citrus species, various molecular techniques have been reported such as random amplified polymorphic DNA (RAPD, [13, 14, 17, 26]), restriction fragment length polymorphisms (RFLP, [6]), amplified fragment length polymorphism (AFLP, [12, 25]), sequence-related amplified polymorphism (SRAP, [3]), start codon targeted (SCoT) polymorphisms [9], inter-simple sequence repeats (ISSRs, [28–30]), LTR-IRAP, LTR-REMAP [5], and SSR [3, 4, 7, 8, 12, 16, 20, 30, 32].

Most of the sweet orange accessions showed a narrow genetic basis [24, 25]. It is suggested that the observed morphological polymorphism must be associated with somatic mutations, which were not detected by some molecular markers.

SSR marker is known as a reliable genetic marker for genetic variation assay and fingerprinting in sweet oranges (*C. sinensis*) cultivars along with several tangerines (*C. reticulata* Blanco, [7, 8]). On the other hand, the homoplasy of SSR microsatellites showed that SSR markers rate in DNA fragments by size. Using characterized amplified polymorphism (CAPS) markers in plant species is a suitable method to reveal sequence variations without using cost-consuming sequencing methods [15].

The present study aims are as follows: (1) genetic fingerprinting of sweet orange (*C. sinensis*) cultivars using SSR molecular markers and (2) evaluation of monomorph allele sequences by using CAPs-SSR method.

## Methods

### Plant materials

The fresh leaves of 80 trees from 13 different cultivars were collected from Mazandaran province during 2018–2019. The studied cultivars and their locations are given in Table 1.

**Table 1:** Studied cultivars, their locations, latitude, and longitude

Cultivar no.	Cultivars	Location	Tree no.	Latitude	Longitude
1	Valencia	Sari	1–4	36.56	53.05
2	Thomson Novel	Sari	5–8	36.56	53.05
3	Cara Cara	Sari	9–12	36.56	53.05
4	Local orange	Sari	13–15	36.56	53.05
5	Sangrin navel	Sari	16–18	36.56	53.05
6	Beirut	Sari	19–22	36.56	53.05
7	Jadeh Nezami	Ghaemshahr	23–32	36.46	52.86
8	Fereydoon kenar	Fereydoon kenar	33–39	36.68	52.52
9	Behshar	Behshar	40–49	36.69	53.54
10	Jadeh Ghadim 1	Ghaemshahr	50–51	36.46	52.86
11	Jooybar	Fereydoon kenar	52–60	36.68	52.52
12	Baed az bazar	Jooybar	61–63	36.63	52.9
13	Jadeh Ghadim 2	Ghaemshahr	64	36.46	52.86

### DNA extraction and molecular marker assay

Genomic DNA was extracted based on CTAB method with some modifications [10, 19]. DNA qualification was checked by using 0.8% agarose gel electrophoresis.

In present study, five high polymorphic SSR loci were used to investigate the genetic diversity and fingerprinting of orange accessions (Table 2). These loci were selected based on a comprehensive study by Liu et al. [16] and verified by alignment at the NCBI information database.

**Table 2:** SSR loci primers used on sweet orange genotype studied. The primer sequences are based on Liu et al. [16]

Sequence	Repeat motif	Length	Forward (5′-3′)	Reverse (5′-3′)	GC	TM
TAA27	TAA	21	GGATGAAAAATGCTCAAAATG	TAGTACCCACAGGGAAGAGAGC	57%	55.69–64.21 °C
CAC15	CAC	22	TAAATCTCCACTCTGCAAAAGC	GATAGGAAGCGTCGTAGACCC	40%	53.11–61.76 °C
AG14	AG	20	AAAGGGAAAGCCCTAATCTCA	CTTCCTCTTGCGGAGTGTTC	55%	54.25–62.72 °C
CAT01	CAT	20	GCTTTCGATCCCTCCACATA	GATCCCTACAATCCTTGGTCC	50%	52.73–61.33 °C
TC26	TC	20	CTTCCTCTTGCGGAGTGTTC	GAGGGAAAGCCCTAATCTCA	50%	51.87–60.61 °C

DNA amplification was carried out in 20 μl reactions containing 12 ng of template DNA, 1.15 μM each of forward and reverse primers, and 10 μl of 1X Master Mix (ParsTous Biotech, Iran).

Thermal program consisted of 94 °C for 5 min; 38 cycles of 94 °C for 40 s, with annealing temperatures for each locus; TAA27 for 52 °C, CAC15 for 52 °C, AG14 for 58 °C, CAT01 for 53 °C, and TC26 for 55 °C for 40 s; and extension segment at 72 °C for 1 min. Final extension was at 72 °C for 5 min. PCR reaction was performed by using Techne Prime thermocycler.

The PCR products were visualized on 3% high-resolution ultrapure agarose gel (UltraPure™ Agarose, Invitrogen, Iran).

For CAPS-SSR assay, *Bam*HI restriction enzyme was used due to the high abundance of its site in the genome. For that, monomorph SSR-PCR products (130, 140, 160, and 180 bps for TAA27; 140, 150, and 170 bps for TC26; 140, 150, 180, 200 bps for AG14; 150, 170, 200 bps for CAT01; and 160, 180, 190, and 200 bps for TC26 locus) were mixed with 0.4 μl of 10 $U\left/\mu l\right.$ Thermo *Bam*HI enzyme (Generay, China), 0.5 μl of the buffer, and 4.5 μl of distilled water. The reaction was incubated at 37 °C for 8–10 h. Digestion products were visualized on 3% UltraPure Agarose gel.

### Data analysis

Each band was scored as present (1) or absent (0). Genetic parameters such as polymorphism percentage (P%), Nei genetic diversity (He), Shannon index (I), and AMOVA test were estimated by using GenAlEx 4.6 software. We used different clustering methods for grouping of the studied genotypes. These dendrograms were constructed by PAST software ver 3.01. Details of genetic grouping by the SSR markers were studied by constructing a heatmap plot. The genetic admixture and assignment of the studied citrus trees were determined by DAPC (discriminant analysis of principal components). These analyses were performed in R package 4.1.

## Results

### SSR amplification

**Fig. 1:**
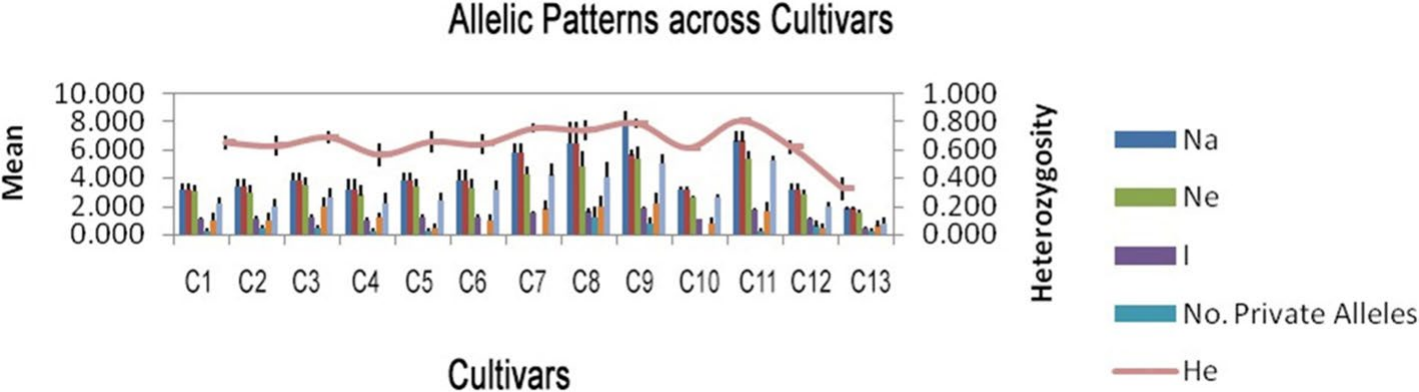
Band pattern of studied cultivars and genetic parameters obtained for each cultivar. Cultivar number according to Table 1. Na, no. of different alleles; Ne, no. of effective alleles = 1/(Sum pi^2); I, Shannon’s information index = −1* Sum (pi × Ln (pi)); He, expected heterozygosity = 1 — Sum pi^2

In total, 5 SSR loci produced 84 alleles ranging from 100 to 300 bps (Fig. S1, S2). The lowest and highest number of alleles were observed in CAC15 and TAA27 loci respectively. All loci produced polymorphic bands (Fig. 1). The highest number of alleles (Na) and effective allele belonged to Behshahr cultivar (no. 9).

The highest and lowest observed heterozygosity (Ho) belonged to cultivars Fereydoon kenar (no. 8) and Jadeh Ghadim 2 (no. 13) with a mean of 0.45 and 0.067, respectively.

**Fig. 2:**
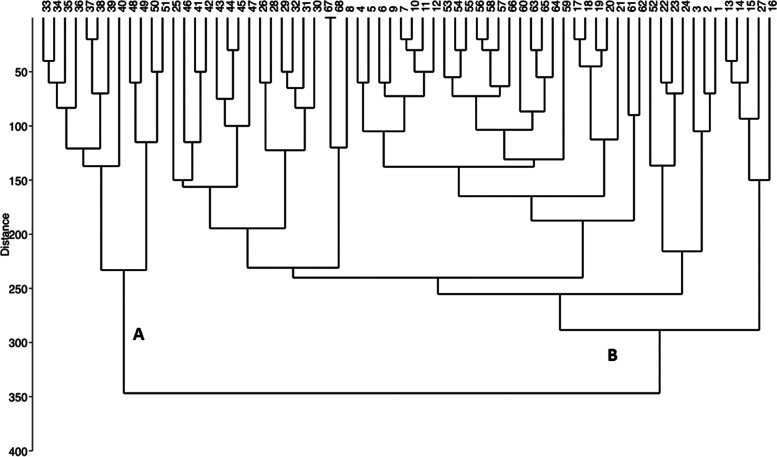
WARD grouping method based on SSR data, cultivars are divided into two main groups: A and B. Number of individuals are according to Table 1

Based on Nei’s genetic distance, the highest distance was observed between Behshahr cultivars (no. 9) and local orange (no. 4). According to the Ward clustering (Fig. 2), genotypes were divided into 2 main groups. Group A includes Thomson Novel, local orange, Cara Cara orange, Jooybar, Sangrin navel and Baed az bazar, Valencia, Jadeh Ghadim 2, and Jadeh Nazami cultivars, while group B includes Baed az bazar, Jadeh Ghadim 1, Beirut, Jadeh Nazami, Jooybar and Behshahr, Beirut, and Fereydoon kenar.

The AMOVA analysis showed that 70% of variance attributed among individuals and groups, while 30% of variance attributed within individuals. This analysis revealed a significant difference between groups (cultivars, *P*-value = 0.001) with *F*_st_ = 0.110. (Table S1, S2).

**Fig. 3.**
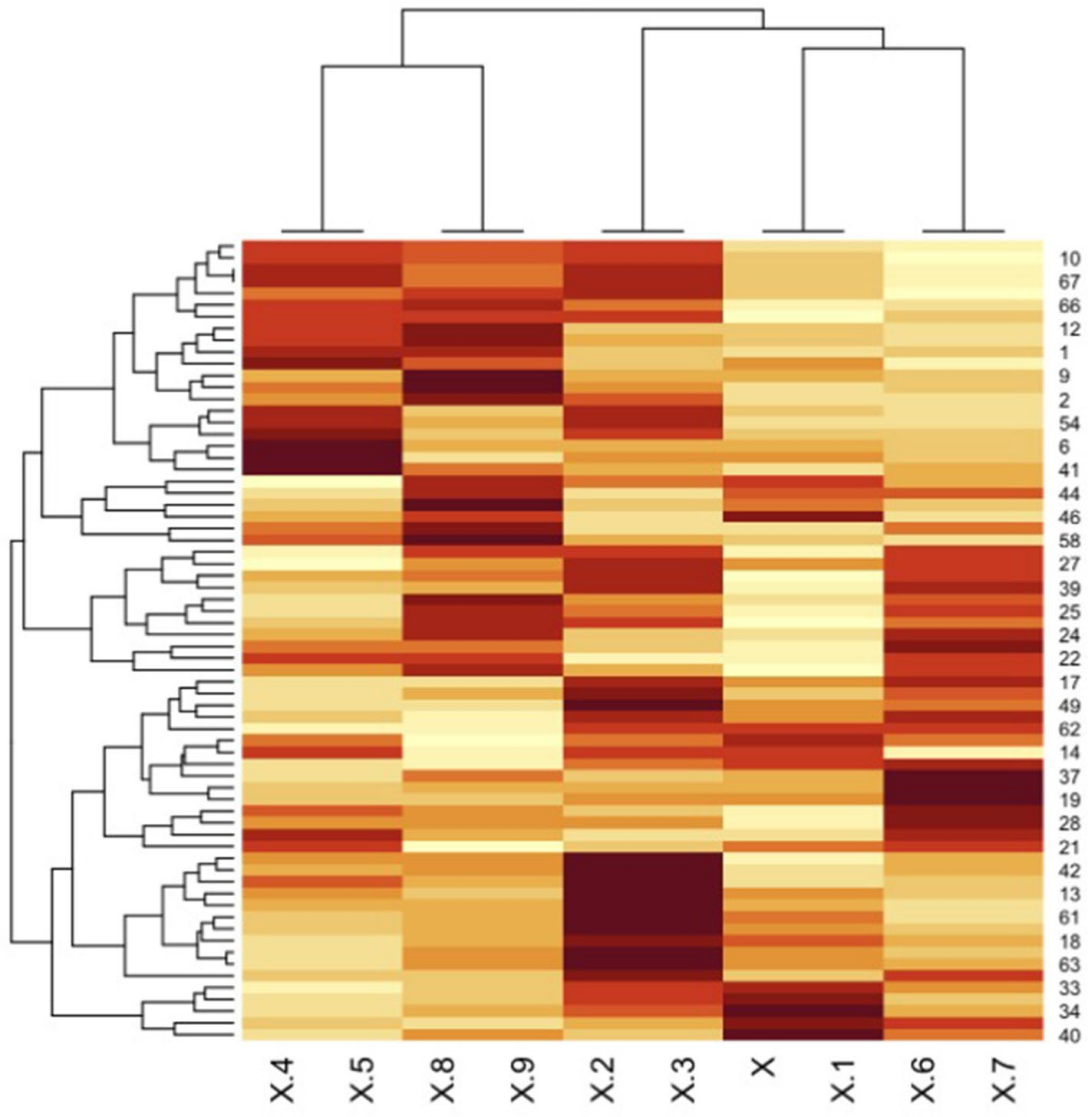
Cluster based on heatmap, analysis based on SSR data, horizontal axis representing the studied loci and vertical graph of the number of cultivars studied according to Table 1

The heatmap plot showed cultivars genetic affinity and the SSR loci which group the cultivars alike to each other (Fig. 3). In heatmap analysis, areas with dark color indicate the high resolution of SSR alleles.

**Fig. 4:**
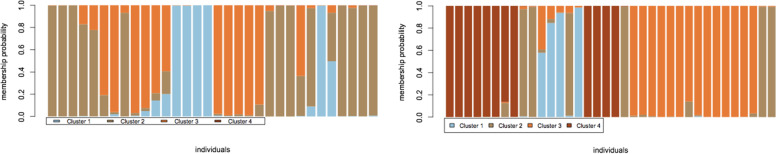
DAPC diagram of genetic structure of sweet orange cultivars based on SSR data. Number of individuals is based on Table 1

The assignment test as illustrated in DAPC plot (Fig. 4) showed close genetic affinity of the studied sweet orange trees due to genetic admixture. In general, the studied genotypes were grouped in four genetic groups. Some of the trees within these cultivars were genetically similar to the other genotypes. This may be due to either mislabeling of the cultivars by locals or due to extensive genetic admixture of the plants.

**Fig. 5:**
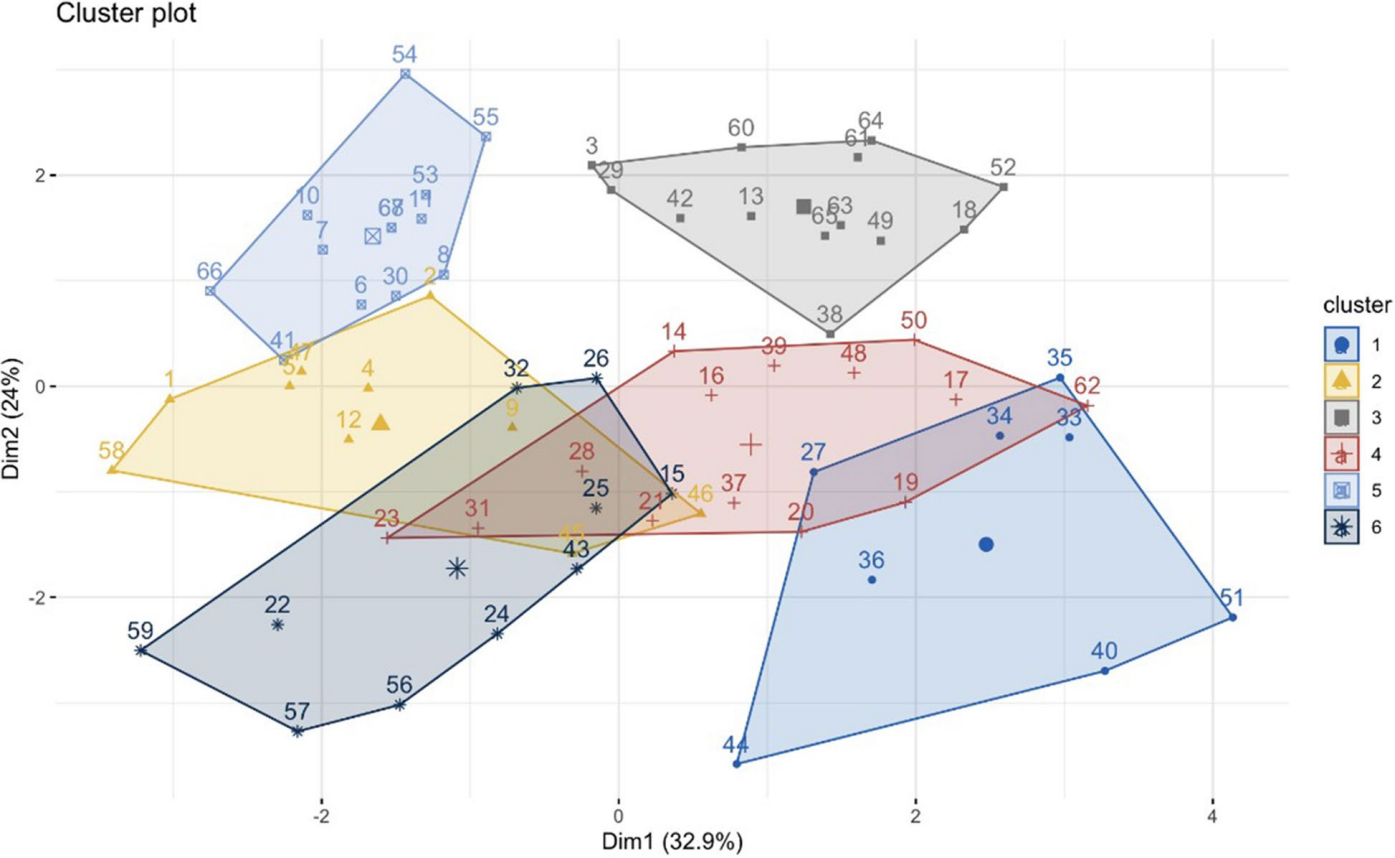
K-means cluster based on SSR alleles. Each cluster is showing with a color*.* Number of individuals are according to Table 1

K-means cluster also showed high admixture of sweet orange trees. Although high similarity has been reported between cultivars, genetic variations were observed among trees of each cultivar (Fig. 5).

Based on Ward dendrogram, the heatmap clustering and k-means clustering results, we considered four major genetic groups for the studied sweet orange cultivars. K-means clustering also shows some degree of overlaps if more than four groups are taken.

### CAP-SSR assay

**Fig. 6.**
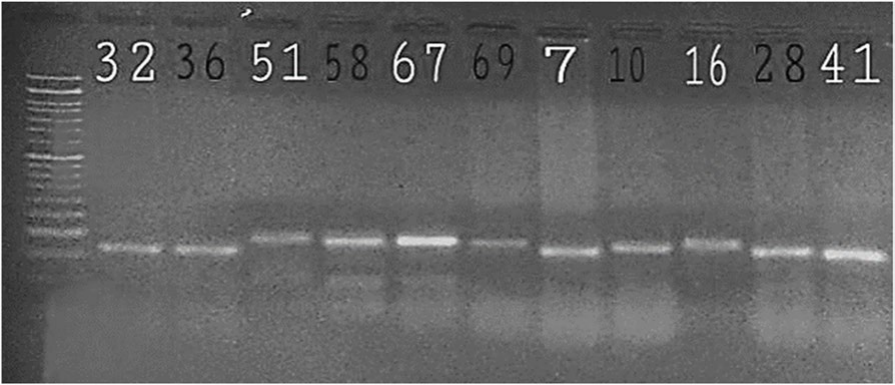
Three percent UltraPure Agarose gel belonging to monomorph alleles after digestion (CAPS-SSR). Ladder 50 bp was used

**Fig. 7.**
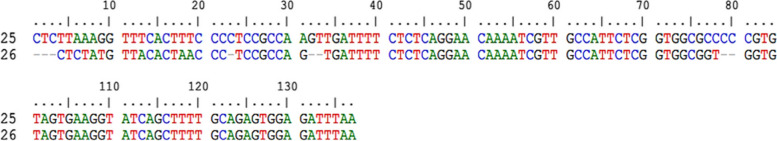
Alignment of two sequences of the monomorph alleles of CAC15 locus

CAPS-SSR (Fig. 6) and alignment of some sequences of the CAC15 locus (Fig. 7) were also investigated for allele homoplasy. The results showed that although products had the same locus in length, they were different in sequences. These data may indicate that studied sweat orange trees which are grouped close to each other may still differ genetically in details of sequences. Therefore, these difference adds up to genetic variability present in citrus germplasm.

## Discussion

The present study showed almost low degree of genetic variability among orange genotypes studied. It has been stated that morphological difference in sweat orange cultivars is due to the occurrence of the somatic mutations in the ancestral trees; hence, orange cultivars though show some degree of morphological variation but have a high level of genetic similarity [11].

The polymorphism percentage of this study was different from studies that were reported by different countries [6, 17] or even in Iran [9]. This controversial differences may come from either nature of markers used or type and number of studied cultivars. However, the PIC value in current study (*PIC* = 0.76) was almost the same with Golein’s study (*PIC* = 0.7 [7]. In general, high genetic similarity in SSR genetic fingerprinting of sweat oranges was also reported by others [21].

The earlier studies performed on the rate of polymorphism in different molecular markers, utilized in genetic fingerprinting of sweat oranges [8, 12], indicate that relatively a higher level of polymorphism is present in SSR markers. For example, Kumar Biswas et al. [12] investigated genetic diversity of thirty-four citrus genotypes from the National Citrus Breeding Center of China by different molecular markers like AFLP, SSAP, SAMPL, and SSR, and reported that SSRs show a higher polymorphism rate compared to the other markers studied. They obtained the mean polymorphism value of 98.46%. In a similar study on the sweat orange genotypes of Iran, Jannati et al. [8] used 15 SSR loci and reported the high polymorphism percentage for three loci: CAT01, TAA27, and AG14.This high level of SSR marker polymorphisms is expected to be due to amplification slip. The codominant common nature of SSR markers also allows the detection of large numbers of alleles in each location and contributes to higher levels of expected heterozygosity [12]. Genetic parameters like *He*, Shannon index, and *Ne* also supported high heterozygosity among trees studied. Therefore, it can be concluded that the SSR marker may be more useful for studies of genome segregation and mapping in citrus than other markers.

The present study revealed a high degree of genetic admixture in sweet orange trees, and individuals studied from different cultivars were genetically similar. This may be partly due to the presence of synonyms, homonyms, and mislabeling within citrus germplasm. It seems that mislabeling is almost a common phenomenon in many cultivated plant species like fig [1], grapevine [5], and grape ([1].

Analysis of SSR loci are based on size of DNA fragments. Length variation is usually the only and most obvious criteria for describing allelic diversity [15]. This may indicate that although monomeric alleles do not appear to be distinct in individuals, they have different sequences.

In order to investigate the homoplasy of microsatellite alleles, CAPs method was performed on monomorphic alleles in cultivars studied. The first report on CAPSs markers was on Arabidopsis [31]. Since then, this method has been repeatedly adapted and used in different plants with different changes to suit specific plant species. Therefore, CAPs have important applications in the analysis of genetic and phylogenetic polymorphisms, especially in closely related species [27, 31]. Therefore, it can be concluded that although a locus has the same allele and size, the sequences are different. Our finding also showed variation among monomorph SSR alleles which may helpful to differentiate genotypes and provide more polymorphism among sweet oranges with high similarities.

## Conclusion

According to present study, the sweet orange genotypes were divided into two main groups. However, the genotypes were genetically very similar due to genetic admixture. In general, all SSR loci used in this study showed high levels of polymorphism (mean 98.46%), which confirmed the high genetic diversity of sweet orange trees in different genotypes in the northern part of the country. Sequencing and CAPS-SSR studies have also provided more variation among monomorph alleles of the same locus can be sequentially different. SSR and CAPs could be helpful for differentiation of sweet orange genotypes.

## Supplementary Information

**Additional file 1: Table S1**. AMOVA table based on SSR alleles for 13 cultivars. **Table S2**. Pairwise groups Fst value between cultivars. **Fig. S1**. Allele patterns of CAC15 SSR locus. 50bps Ladder. **Fig. S2**. Allele patterns of TAA27 SSR locus. 50bps Ladder.

## Data Availability

Raw data are available in request.

## Abbreviations

CAP: Characterized amplified polymorphism
CTAB: Cetyltrimethylammonium bromide
DAPC: Discriminant analysis of principal components
SSR: Short simple repeat
