# Supplementary material for: A new insight on genetic diversity of sweet oranges: CAPs-SSR and SSR markers
Source: J Genet Eng Biotechnol. 2022 Jul 14;20:105. doi: 10.1186/s43141-022-00393-6 (PMC9283602; doi:10.1186/s43141-022-00393-6)
Supplement: Supplementary file 1 — Additional file 1: Table S1. AMOVA table based on SSR alleles for 13 cultivars. Table S2. Pairwise groups Fst value between cultivars. Fig. S1. Allele patterns of CAC15 SSR locus. 50bps Ladder. Fig. S2. Allele patterns of TAA27 SSR locus. 50bps Ladder. [file 43141_2022_393_MOESM1_ESM.docx]

Table S1 AMOVA table based on SSR alleles for 13 cultivars.

| Source | df | SS | MS | Est. Var. | % |  |  |  |
| --- | --- | --- | --- | --- | --- | --- | --- | --- |
| Among Pops | 12 | 74.439 | 6.203 | 0.250 | 11% |  |  |  |
| Among Indiv | 62 | 209.267 | 3.375 | 1.351 | 59% |  |  |  |
| Within Indiv | 75 | 50.500 | 0.673 | 0.673 | 30% |  |  |  |
| Total | 149 | 334.207 |  | 2.274 | 100% |  |  |  |
|  |  |  |  |  |  |  |  |  |
| F-Statistics | Value | P(rand >= data) | |  |  |  |  |  |
| Fst | 0.110 | 0.001 |  |  |  |  |  |  |
| Fis | 0.667 | 0.001 |  |  |  |  |  |  |
| Fit | 0.704 | 0.001 |  |  |  |  |  |  |
|  |  |  |  |  |  |  |  |  |
| Fst max | 0.192 |  |  |  |  |  |  |  |
| F'st | 0.573 |  |  |  |  |  |  |  |
|  |  |  |  |  |  |  |  |  |
| Nm | 2.026 |  |  |  |  |  |  |  |
|  |  |  |  |  |  |  |  |  |
| Probability, P(rand >= data), for Fst, Fis and Fit is based on standard permutation across the full data set. | | | | | | | | |
|  |  |  |  |  |  |  |  |  |
| Fst = AP / (WI + AI + AP) = AP / TOT | | |  |  |  |  |  |  |
| Fis = AI / (WI + AI) |  |  |  |  |  |  |  |  |
| Fit = (AI + AP) / (WI + AI + AP) = (AI + AP) / TOT | | | |  |  |  |  |  |
| Nm = [(1 / Fst) - 1] / 4 | |  |  |  |  |  |  |  |
| Key: AP = Est. Var. Among Pops, AI = Est. Var. Among Individuals, WI = Est. Var. Within Individuals | | | | | | | | |
|  |  |  |  |  |  |  |  |  |

Table S2. Pairwise groups Fst value between cultivars

| c1 | c2 | c3 | c4 | c5 | c6 | c7 | c8 | c9 | c10 | c11 | c12 | c13 |  |
| --- | --- | --- | --- | --- | --- | --- | --- | --- | --- | --- | --- | --- | --- |
| 0.000 |  |  |  |  |  |  |  |  |  |  |  |  | c1 |
| 0.174 | 0.000 |  |  |  |  |  |  |  |  |  |  |  | c2 |
| 0.165 | 0.159 | 0.000 |  |  |  |  |  |  |  |  |  |  | c3 |
| 0.212 | 0.204 | 0.140 | 0.000 |  |  |  |  |  |  |  |  |  | c4 |
| 0.146 | 0.165 | 0.113 | 0.163 | 0.000 |  |  |  |  |  |  |  |  | c5 |
| 0.201 | 0.157 | 0.178 | 0.183 | 0.138 | 0.000 |  |  |  |  |  |  |  | c6 |
| 0.133 | 0.106 | 0.111 | 0.184 | 0.121 | 0.115 | 0.000 |  |  |  |  |  |  | c7 |
| 0.132 | 0.147 | 0.140 | 0.196 | 0.147 | 0.171 | 0.100 | 0.000 |  |  |  |  |  | c8 |
| 0.103 | 0.126 | 0.127 | 0.187 | 0.122 | 0.135 | 0.077 | 0.072 | 0.000 |  |  |  |  | c9 |
| 0.185 | 0.224 | 0.194 | 0.243 | 0.178 | 0.170 | 0.161 | 0.143 | 0.100 | 0.000 |  |  |  | c10 |
| 0.109 | 0.132 | 0.126 | 0.179 | 0.109 | 0.124 | 0.057 | 0.074 | 0.046 | 0.112 | 0.000 |  |  | c11 |
| 0.199 | 0.134 | 0.147 | 0.231 | 0.132 | 0.161 | 0.121 | 0.148 | 0.151 | 0.222 | 0.120 | 0.000 |  | c12 |
| 0.240 | 0.317 | 0.301 | 0.358 | 0.313 | 0.334 | 0.277 | 0.268 | 0.266 | 0.348 | 0.250 | 0.323 | 0.000 | c13 |
|  |  |  |  |  |  |  |  |  |  |  |  |  |  |
|  | | |  |  |  |  |  |  |  |  |  |  |  |


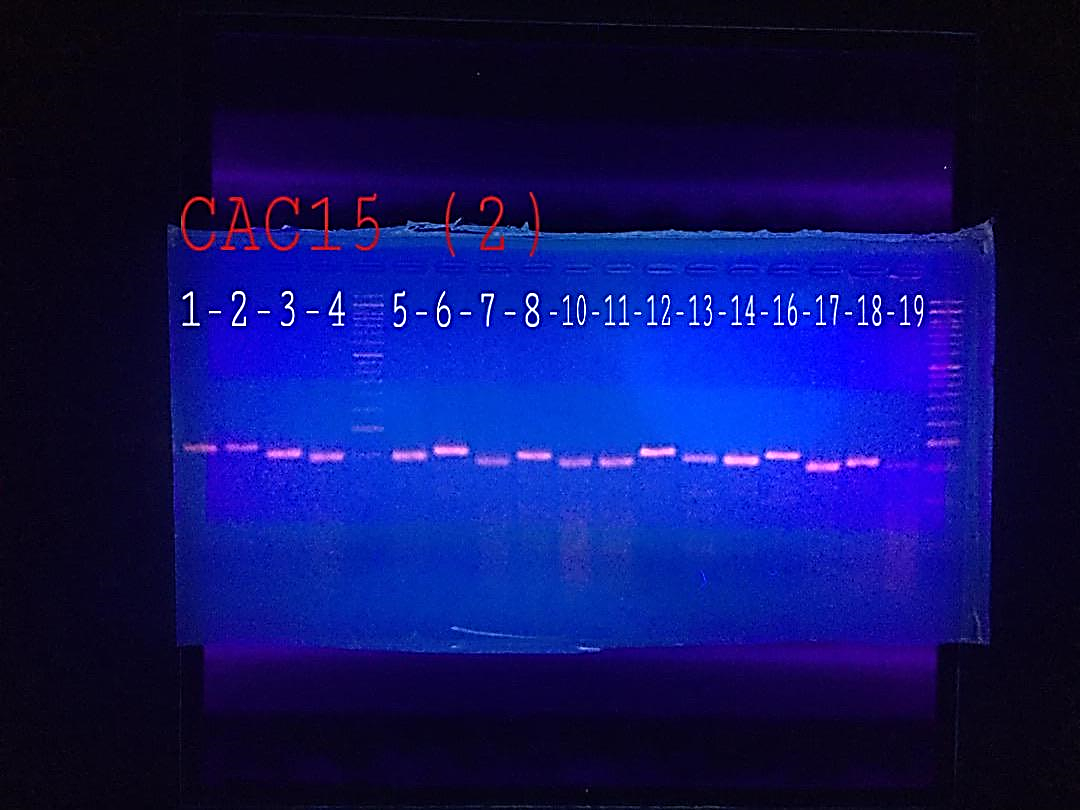


Fig S1. Allele patterns of CAC15 SSR locus. 50bps Ladder


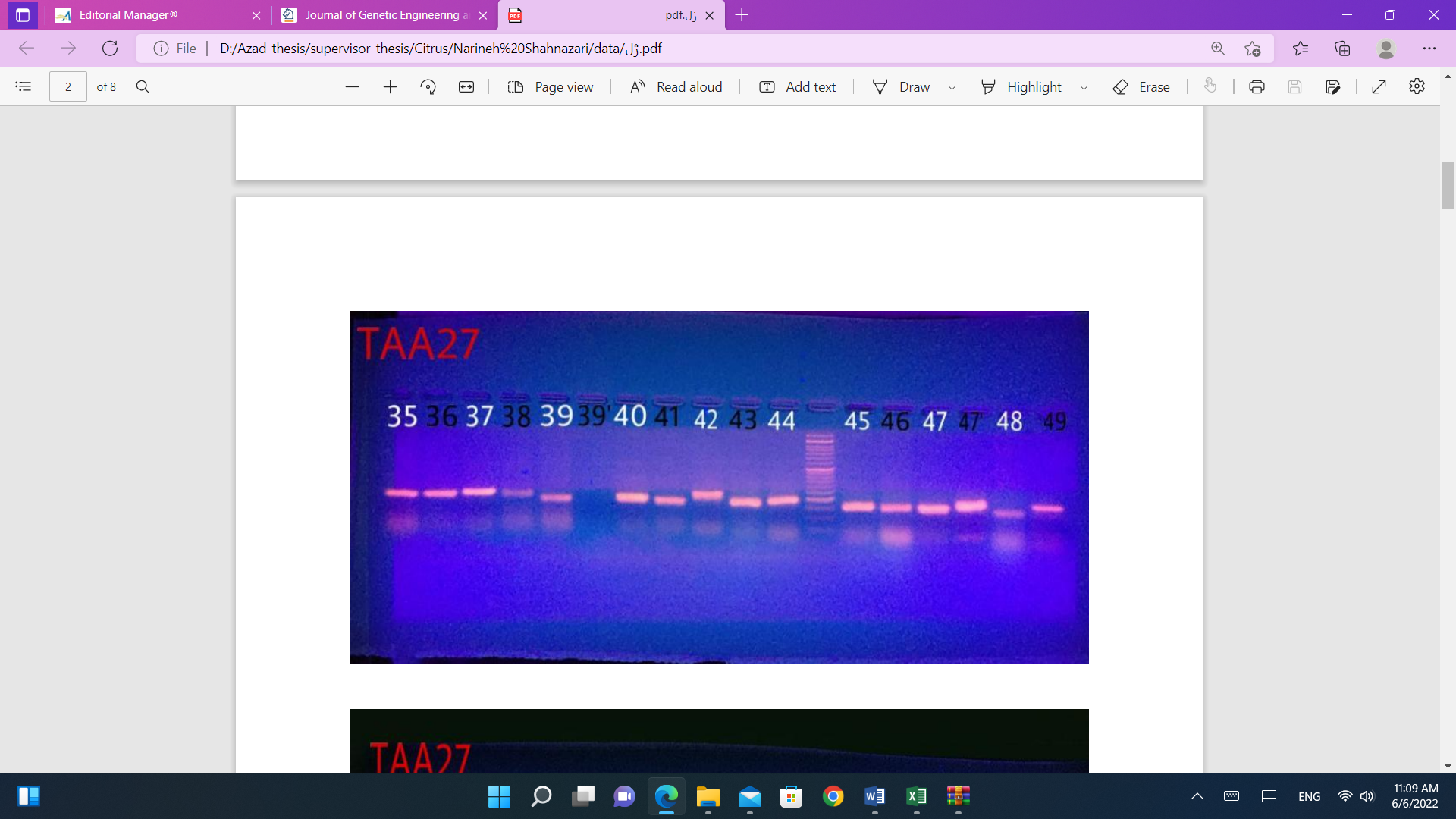


Fig S2. Allele patterns of TAA27 SSR locus. 50bps Ladder
